# Factors affecting healthcare pathways for chronic lung disease management in Vietnam: a qualitative study on patients’ perspectives

**DOI:** 10.1186/s12889-021-11219-4

**Published:** 2021-06-15

**Authors:** Thu-Anh Nguyen, Yen Ngoc Pham, Nhung Phuong Doan, Thao Huong Nguyen, Toan Thanh Do, Giap Van Vu, Guy B. Marks, Shannon McKinn, Joel Negin, Sarah Bernays, Greg J. Fox

**Affiliations:** 1 Woolcock Institute of Medical Research, 298 Kim Ma, Ba Dinh, Hanoi, Vietnam; 2 Faculty of Medicine and Health, University of Sydney, Sydney, Australia; 3 School of Preventive Medicine and Public Health, Hanoi Medical University, Hanoi, Vietnam; 4 Bach Mai Hospital, Hanoi, Vietnam; 5 School of Public Health, University of Sydney, Sydney, Australia; 6 London School of Hygiene and Tropical Medicine, London, UK; 7 Australia South Western Sydney Clinical School, University of New South Wales, Sydney, Australia

**Keywords:** COPD, Asthma, Healthcare pathway

## Abstract

**Background:**

Chronic obstructive pulmonary disease (COPD) and asthma rank among the leading causes of respiratory morbidity, particularly in low- and middle-income countries. This qualitative study aimed to explore the healthcare pathways of patients with chronic respiratory disease, and factors influencing their ability to access healthcare in Vietnam, where COPD and asthma are prevalent.

**Methods:**

We conducted 41 in-depth interviews among patients, including 31 people with COPD, eight with asthma and two with asthma-COPD overlap syndrome. Participants were recruited at provincial- or national-level health facilities in two urban and two rural provinces in Vietnam. The interviews were audio-recorded, transcribed, and analysed using thematic analysis.

**Results:**

Patients’ healthcare pathways were complex and involved visits to multiple health facilities before finally obtaining a definitive diagnosis at a provincial- or national-level hospital. Access to healthcare was affected considerably by participants’ limited knowledge of their respiratory conditions, the availability of social support, especially from family members, the costs of healthcare as well as health system factors (including the coverage of public health insurance, the distance to health facilities, and attitude of healthcare providers).

**Conclusion:**

The study demonstrated the need for improved access to timely diagnosis and treatment of chronic lung disease within the lower level of the health system. This can be achieved by enhancing the communication skills and diagnostic capacity of local healthcare workers. Health education programmes for patients and caregivers will contribute to improved control of lung disease.

## Backgrounds

Chronic obstructive pulmonary disease (COPD) and asthma are chronic respiratory diseases (CRDs) that are responsible for over 3.5 million deaths annually [1, 2]. While COPD is characterized by chronic irreversible obstruction of lung airflow [1], asthma is characterized by reversible airflow limitation that causes recurrent wheeze, shortness of breath and chest tightness [3]. With inadequate preventive therapy, severe exacerbations can occur [4], leading to a reduced quality of life and increasing the risk of death [1, 2]. Therefore, appropriate treatment and management for CRDs is critical to relieve respiratory symptoms and decrease the number of attacks.

Despite their importance, these obstructive lung diseases are under-diagnosed and under-treated in many settings [1, 2, 5, 6]. This is often due to a lack of awareness about the diseases, different definitions used for diagnosis, limited capacity to perform spirometry, a lack of clear and practical diagnosis guidelines and poor access to health care. These challenges are particularly common in low and middle income countries (LIMC) [7, 8]. Indeed, research suggests that around 90% of COPD-related deaths and 80% of asthma-related deaths occur in LMIC [1, 2, 9].

Vietnam is a LMIC in Southeast Asia where obstructive lung diseases are commonly undiagnosed. Similar situation has been found in other countries in Asia [7, 10]. The prevalence of COPD is between 7 and 10% [11–13], and asthma prevalence is estimated to be 5.6% [14]. Several studies found that patients often present to higher levels of health facilities before obtaining an accurate diagnosis, then have a delayed diagnosis and suffer from preventable exacerbations, increasing the economic burden to individuals and the health system [8]. This could be explained by several health system barriers such as, a lack of knowledge about the optimal pathways for diagnosis and treatment of these diseases among Vietnamese doctors [12, 13, 15–17], complex treatment guidelines, and limited availability of medications in primary care settings (district and commune level) [15, 18, 19]. In 2010, the Vietnamese Government established a National Project for the Prevention and Control of COPD and asthma to increase early diagnosis and management of CRDs in the community and introduced a Practical Approach to Lung health (PAL) programme [20]. Joint funding from these two initiatives was used to establish Chronic pulmonary disease Management Units (CMU) which aim to address these health system challenges and improve management of COPD and asthma within primary healthcare [21]. Although there have been some improvements in access to diagnosis and treatment for CRDs in the community, substantial detection gaps persist. Therefore understanding the barriers to quality health care from perspective of patients with CRD in Vietnam could help to address to these gaps.

This qualitative study aimed to explore healthcare pathways among patients with CRD in the Vietnamese healthcare system; and to identify factors influencing the healthcare seeking behaviour from the perspective of patients. Study findings were used to inform the design of an intervention to enhance access to standardized therapy for COPD and asthma within the lower levels of the health system in Vietnam.

## Methods

### Setting

Vietnam is a country of 96 million people, with a healthcare system divided by hierarchy into four administrative levels: national level – the highest level – as well as provincial, district and commune levels [22]. Lower level (district and commune) health facilities may refer patients to the higher level (national and provincial) health facilities for specialized diagnostic tests or treatment. Patients may also self-refer to health facilities at the higher level if they are able to afford the out-of-pocket costs for medical services. Health insurance coverage is approximately 85% [23]. People insured could visit any district or commune health facilities. However, health facilities at provincial and national level would require a referral letter.

This qualitative study is a part of the Vietnam COPD, Asthma and Prevention of Smoking (VCAPS) study, a programme of research that aims to characterize and address the barriers to effective care for CRDs in Vietnam (the trial registered number is ACTRN12620000649910). A qualitative study design was used because it is an appropriate method to explore patients’ experiences of the care-seeking pathways. Individual in-depth interviews were used to identify and understand the specific and local contextual factors that shaped participants’ journeys to diagnoses and care. Four provinces were selected, to represent the variations in population size and geographical location. They comprised the two most populous cities in the north (Hanoi) and south (Ho Chi Minh City) as well as two rural provinces (Thanh Hoa in the north, and Ca Mau in the south). In these settings, six [6] national and provincial hospitals providing care and treatment for CRDs patients were selected to recruit study participants.

### Participant selection

Participants aged 18 and over, diagnosed with either COPD or asthma at inpatient or outpatient departments of participating high-level hospitals, were eligible to participate. Purposive sampling was employed to identify suitable participants with a range of personal characteristics (including age, gender, residence in urban or rural settings and type of diagnosis). At the hospital, health workers identified eligible participants, and arranged an appointment for researchers to conduct an interview.

### Data collection

Semi-structured individual interviews were conducted by four Vietnamese researchers between January and September 2017 using a flexible topic guide (Supplementary). Key topics were identified from the existing literature and sought to elicit participants’ descriptions of their personal care-seeking experiences. All researchers had experience conducting qualitative interviews and were trained to be familiar with the research topic. Following the topic guide, participants were asked about their individual healthcare seeking process, perceived facilitators and barriers in accessing healthcare for their conditions, CRD-related knowledge and attitudes, experiences in the use of healthcare services, and factors affecting their decisions in selecting health facilities/services. Interviews were conducted with individually and in a private setting, with the exception of one interview in which the participant was accompanied by a family member. Interviews lasted about 35 to 90 min. Twenty one out of the 41 participants consented for the interview to be audio-recorded. Interviews were transcribed by trained transcribers. Interviews with the 20 participants who did not agree to audio-recording were documented with detailed notes during and after the interview. All transcripts and fieldwork notes were anonymised to ensure the confidentiality of the participants.

### Data analysis

Interview transcripts and fieldwork notes were analysed using thematic analysis. Four qualitative researchers familiarised themselves with the dataset by reading the transcripts and notes multiple times. Through discussion a coding framework was developed, which was then applied line-by-line to the dataset [24, 25] and recurring codes, as well as how a nexus of codes clustered around particular stages or experiences, were noted. The coded data were charted together to support thematic interpretation. This analytical process was informed by ongoing analytical discussions among the research team, including the drafting, sharing and refining of analytical memos about emerging themes [26]. Particular attention was paid to the similarities and differences across participants’ data to generate an explanation of the findings, including considering the learning from any deviant cases, as is appropriate to ensure the trustworthiness and rigour of qualitative analysis [27].

**Fig. 1:**
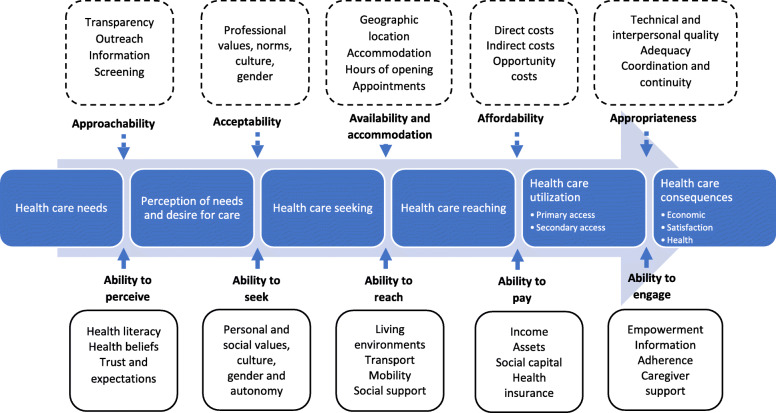
Conceptual framework of access to care (Levesque et al. (2013))

The thematic framework that was derived through this analytical process had clear resonance with the conceptual framework developed by Levesque et al. regarding the abilities of patients accessing healthcare services [28], however it was not a model which framed the research a priori but instead was considered at a later stage of the analysis to guide further analysis on patients’ health-seeking pathways for CRD patients, as well as the existing barriers to care perceived by these health service users. The pertinence of the Levesque framework is that it incorporates the various stages of the healthcare-seeking pathways, serving as a productive lens through which the accounts of the study participants can be examined. Figure 1 illustrates how these factors influence the health-seeking pathways of the patients. The top of the figure presents five factors from the perspectives of those working within the health system (in the dotted-boxes); including, approachability, acceptability, availability and accommodation, affordability, and appropriateness. The bottom of the figure presents five factors reflecting the patient’s experience of interacting with the ‘health system’ and how this shapes their access to care. In this study, we focus on the patient’s abilities to access and interaction with the healthcare system.

Within this framework, *‘Ability to perceive’* refers to knowledge about health conditions which enables patients to identify their health needs, as well as patients’ knowledge and attitude in relation to their own health and illnesses [28]. *‘Ability to seek’* specifies patients’ awareness of available services, as well as their autonomy and capacity to seek care which can be influenced by personal, cultural and social factors such as gender [28]. *‘Ability to reach’* conveys patients’ ability to access healthcare physically, including factors like transportation or the flexibility of their occupation [28, 29]. The ‘*ability to pay’* refers to the ability to generate financial resources to pay for healthcare services without incurring catastrophic expenditures [28]. Finally, *‘ability to engage’* describes the participation and involvement of patients in their treatment process, such as how involved they are in decisions regarding their illness and treatment [28, 29].

### Ethical approvals and considerations

The study conducted is in accordance with the Declaration of Helsinki guideline. Ethic approvals were obtained from the Human Research Ethics Committee (HREC) of the University of Sydney, application reference number 2017/736 and Bach Mai Hospital, Vietnam, decision number 2381 QD/BM. A written inform consent was obtained from all interviewees before every interview.

## Results

### Participant characteristics

Interviews were conducted with individually and in a private setting, with the exception of one interview in which the participant was accompanied by a family member. Characteristics of participants are presented in the Table 1.

**Table 1:** Characteristics of the study participants

Participant characteristics (***n*** = 41)	
Age (years)	68 (median)
Gender	n (%)
Male	30 (73)
Female	11 (27)
Diagnoses	n (%)
COPD	31 (75.6)
Asthma	8 (19.53)
Asthma-COPD overlap syndrome (ACOS)	2 (4.87)
Provinces	n (%)
Hanoi	7 (17)
Thanh Hoa	11 (26.8)
Ca Mau	10 (24.4)
Ho Chi Minh city	10 (24.4)
Other provinces	3 (7.4)
Department where the patients are being treated/followed up	n (%)
Inpatient department	13 (32)
Outpatient department	28 (68)

### Healthcare seeking pathways of COPD/asthma patients in Vietnam

We found that healthcare seeking pathways among CRD patients were complex, with the involvement of health facilities at different levels in both public and private sectors. Many participants reported mistaking their initial respiratory symptoms for ‘normal’ smoking-related effects, or the symptoms of flu or bronchitis. These assumptions led to self-medicating using cough remedies and/or antibiotics, either self-prescribed or on advice from pharmacy staff, without medical assessment. When the symptoms appeared, the participants tended to visit primary health care (mostly district hospitals) where they could use health insurance. They often went back and forth between facilities seeking diagnosis and treatment for their ongoing symptoms. When symptoms were more frequent and severe, they went to higher level facilities, with or without referral of the doctors at primary health care facilities, for diagnosis and treatment. While seeking treatment from these health facilities and/or between treatment periods, some participants also reported seeking healthcare services from community pharmacists and private clinics or using traditional remedies in parallel. They often went back and forth between healthcare facilities and tried several times until a diagnosis of the participant’s condition(s) was confirmed, and symptoms were resolved after a few months or years. This usually occurred in provincial or national hospitals, where hospital’s status leads patients to feel the care is satisfactory. Although CRD requires a long-term management approach, participants often stopped taking treatment once their acute symptoms had resolved. They then re-entered their complex healthcare pathways when their respiratory symptoms recurred.

To further illustrate the healthcare seeking pathways of COPD/asthma patients in Vietnam we present two cases: one from Hanoi representing participants from the north and in urban areas, and one from Ca Mau – a southern rural province .

#### Example 1

**Fig. 2:**
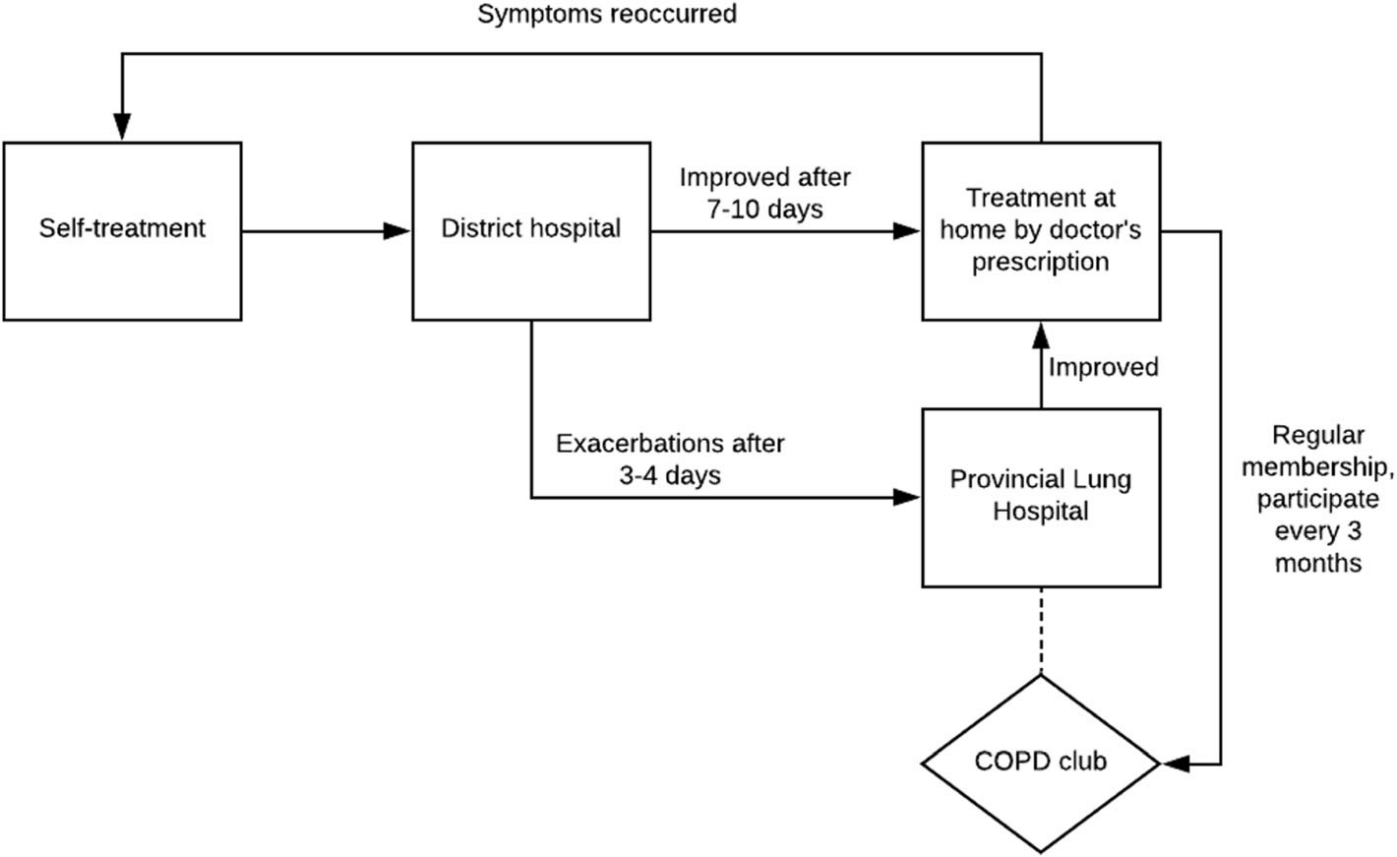
The pathway to get health care service of participant A

Participant A, age 73, was from a rural area of Hanoi. His COPD diagnosis was confirmed 20 years ago, when he was in the military. When he had heightened symptoms (which he described as having dryness in his throat, a persistent cough and shortness of breath), services at the district hospital were sought first, as it was near his home and it was the health facility where his health insurance was registered. If his condition improved, (which he described as finding it “easier to breathe”), he would get a prescription from the district hospital to continue the treatment at home. However, if the symptoms got worse (“I breathed like a sick cat and could not eat for three to four days”), he was referred to a provincial lung hospital. If the situation did not require emergency, he usually chose to stay in the district hospital due to the shorter travel distance and the availability of family support, especially from his children. He did not stop receiving care at the district hospital but moved back and forth between the provincial lung hospital and the local district hospital as the need dictated (Fig. 2).

#### Example 2

**Fig. 3:**
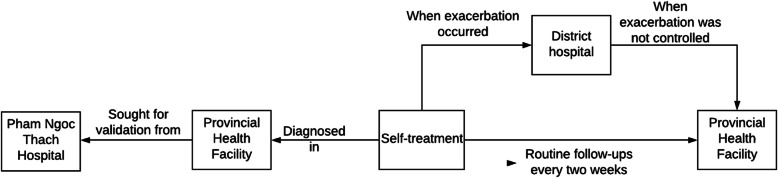
The pathway to get health care service of participant B

Participant B, a 65-year-old woman, was from Ca Mau province in southern Vietnam. After self-medicating for coughing and shortness of breath with antibiotics and traditional medicines was unsuccessful, she chose a provincial-level healthcare centre as the first point of the pathway. This health facility was chosen instead of the district-level hospitals because it was well-known for treating respiratory diseases, despite being far away from her home. Here, she was diagnosed with COPD and was given a prescription which includes inhaled medication to control symptoms. Even though the health facility was trusted by the participant, she still wanted to validate her diagnosis by visiting a tertiary hospital for lung diseases in the large southern city of HCMC. Since the diagnosis made by the tertiary hospital was similar to that of the provincial health centre, she decided to follow the routine treatment in the provincial health facility by visiting once a fortnight. However, she chose to visit the district hospital, which was near her residence, whenever her symptoms increased, due to convenience (Fig. 3).

### Factors influencing patients’ ability to access healthcare

Our thematic analysis demonstrated factors that led to a complex health seeking pathway among CRD patients, and are structured according to the five domains of the Levesque framework [28].

### Ability to perceive

The ability to perceive healthcare needs and appropriate services of CRD patients was influenced mainly by patients’ lack of knowledge about their health condition and health service availability which might restrict their awareness of the appropriate services available to them, and may influence their self-medicating behaviour when symptoms appeared or reoccurred.

### Limited knowledge of condition perpetuated reliance on self-medication

Patients’ understanding about their chronic respiratory illness is critical to ensuring timely access to healthcare services when first symptoms appear, and attacks occur. This knowledge is also important to ensure optimal adherence to appropriate long-term treatments. However, among participants in this study, the ability to perceive their health needs and to access available respiratory disease services was hindered by their limited knowledge about the diseases.

Many participants did not know what their health condition was. They were not given sufficient information and support by their treating doctors to develop an adequate understanding of their condition, even after they had been diagnosed with COPD or asthma. Importantly, having received a diagnosis it was common from participants’ accounts that what this meant and how it could be effectively managed was not adequately conveyed to them in ways that they understood. In particular, most participants did not understand that their condition could not be necessarily resolved through curative treatment and instead may require long-term treatment to prevent exacerbations of their chronic condition. Thus when a symptom reoccurred, patients sought out alternative or escalated care often experiencing a disruption in the continuity of care for their ongoing condition.

*I went to this hospital and they asked me to blow into a lung machine, it meant that they checked my lung function. But they did not explain anything. (Male, 81, Hanoi, COPD).*

*The reality is that all treatment was decided by the doctor [at provincial hospital] and I could not ask a lot. I only asked and knew that I had chronic pneumonia and pulmonary artery blockage. I know that it is a kind of lung disease. So I went to this national hospital so they could find out the right disease for my condition and for better treatment (Male, 72, Thanh Hoa, COPD).*

*At first, I breathed with difficulty. I asked around and when to several private doctors for injection and bought medicines for six months. After that it [breathless] came back. Dr Five at this commune gave me another six months of injection and ten days of oral medicines. He said if the condition got better, I could stop taking medicine. But when it was too serious, I went to ICU at provincial hospital. (Female, 68, Ca Mau, asthma).*

Inadequate knowledge about their conditions and the available treatment options led many participants to self-medicate until the symptoms worsened to the point where they could no longer be managed. As mentioned in the previous sub-theme, many self-medication behaviour often occurred at an early stage in the participants’ health-seeking process, but it also led to long delays in seeking care and reduced opportunities for early intervention. A large proportion of participants reported that they bought medications from community pharmacies without a prescription and had experimented with different remedies that they had heard about anecdotally or by re-using previous prescriptions.

*I have a lung disease, they [doctor] said it is some kind of obstruction. I have had this condition for more than ten years and went to many places [health facilities] until three years ago [doctors at] two hospitals in Ho Chi Minh city found out this disease. Prior to coming to the hospitals, I treated my conditions with traditional medicines, some kind of leaves that I forgot the name with honey. When I was too tired, I went to buy medicines at pharmacy or visited Doctor Six for injection. When I cannot stand, too tired and difficult to breath, I went to hospital that I had good experience or that was referred by my neighbour for examination and treatment. I stopped the treatment when finishing my prescription. No doctor asks me to return. (Male, 73, Ca Mau, COPD).*

*Last year and this year, I have had to go to the hospital. Otherwise, normally, I just take medications. I told [the drug sellers] about my breathlessness, then they sold me asthma medications … This helped at first but now when I start ageing, my condition worsened, then become chronic. (Male, 50, Hanoi, asthma).*

Traditional remedies were the other common form of self-medication. However, instead of going to a traditional medicine doctor, these remedies were referred to the participants by other people based on their experience or word-of-mouth. For example:

*When I went to Laos, the ethnic people there told me to catch chameleons, then burn them, add some honey to make a drink. So, when I was ill, I went to Long Xuyen, Chau Doc, where there are chameleons. I bought around 20 of them. Or I took a toad bile diluted in hot water. Or another trick is to drink water from crushed Water Hyssop together with honey when you lie down; you are not allowed to drink in sitting or standing position. It did help. Other medicines were useless. (Male, 69, Ca Mau, COPD).*

*I used ginger with honey, seahorses pickled wine, or sometime drinking burn coal. Sometimes I used dried sliced lime, star fruit pickled in rock candy. I also took some Chinese traditional pills, but I drop it now. I took it without knowing if it would work or not. (Female, 68, Ca Mau, Asthma).*

### Ability to seek care

#### Trust

Participants’ ability to seek healthcare services was considerably influenced by trust in the health system and at various levels, as well as their own social circumstances. The extent of their trust was moderated by their own (or someone from within their social network’s) previous experiences with the services, the reputation of the chosen health facilities, or from the perception that the higher the level of the health facility, the better the service quality.

For example:

*I chose [this hospital] because I have a nephew who is also working there. He advised me to go there for a health examination. (Male, 60, Hanoi, COPD).*

*I chose [this health facility] because the treatment here is better than [other places], there have also been lots of people receiving treatment here. I have a friend who also has the same condition, he told me to come here. (Female, 65, Ca Mau, ACOS).*

Trust was also augmented when they perceived rapid improvement in their conditions after receiving treatment from the health facilities.

*Thanks to the doctors here … I have had bronchitis for quite a while, but after 3 days in the hospital, my symptoms reduced. What have made me believe in the doctors is that I can see my condition improving considerably in a short time. I feel so relieved and trust in our health system. (Male, 68, Hanoi, COPD).*

In contrast, the trust in a health facility can also be lost after an unpleasant experience from the past, which led to the participants refusing to use health services from that hospital.

*When my dad stayed in [that hospital], he had to remain there almost every month. When he had CT scan, they said his lungs were normal. But when he was breathless, we moved him to [another hospital], they said his lungs were white. Since then, I have disliked [that hospital]. (Female, 39, HCMC, with asthma).*

Moreover, the reputation of the health facilities also attracted people with CRDs. This reputation might arise from participants’ awareness of the wider range of available services, and their perceptions of the higher quality and modern tests, provided in the hospitals, for example:

*Here they have X-ray [facilities], blood tests, sputum tests so that they can make a diagnosis correctly. These things were not available in the commune [health clinic], how can they diagnose my condition there? (Male, 69, Ca Mau, with COPD).*

There was also a common perception among the participants that hospitals at higher level would provide better healthcare services on account of having better capacity to diagnose their conditions and higher quality doctors.

*It is not like I did not trust them. But in general, everyone wants to come here [the higher-level hospital] as there have been some incorrect diagnoses that led to ineffective and costly treatment [in lower-level hospital]. When I come to [this hospital], I can have spirometry. The staff were very nice. But it was so crowded, my number [in the queue] was 42! Probably there are lots of people trusting [this hospital]. (Male, 60, Others, COPD).*

*I think [this hospital] must be better than [the provincial hospital], you know like a university graduate is better than someone who only attended vocational training. (Male, 72, Thanh Hoa, with COPD).*

#### Family burden

Family burdens were mentioned as important barriers to their health-seeking and further, to their health outcomes. This was gendered, with the influence of family responsibilities being given greater emphasis among female interviewees, underscoring the social norms in Vietnamese culture in which looking after the family is considered to be the duty and responsibility of women. In circumstances where there is very limited access to formal support, the responsibility falls to female relatives to provide care for those in the family. This was detrimental for a number female participants, for whom seeking care became secondary to their responsibility to care for others.

*The doctors wanted me to [stay in the hospital], but I told them that there is only me and my husband living together now, no one else so I cannot stay. I keep obtaining medicines to allow treatment at home. My husband is a wounded soldier, his illnesses keeps coming back so I need to take care of him … My children also stay far away … (Female 1, 70, Thanh Hoa, with COPD).*

In some cases this was mitigated by the provision of care from younger relatives. However there were many, often older participants, who felt that they could not call on such support from their families, which left them without anyone to provide the necessary care if hospitalised. This might include a long list of tasks such as providing meals, personal hygiene, etc. Several participants described being afraid to disrupt their children’s lives. Others, described their embarrassment and concern of being judged by if they did not have their children beside them.

*There is only me and my husband, no one else. Who will look after me if I come here? You see, when you come here [to this hospital], it means your condition must be really bad. But when you don’t have anyone going with you, to take care of you, they will laugh at me, like: “Why does she stay in the hospital without anyone looking after her?”. But my children still need to work, my grandchildren are still so small … (Female 2, 70, Thanh Hoa, with COPD).*

*There is just me and my husband. My children are living far away. If it [this hospital] is near, I can ride a bike slowly, but when I must come here [to this hospital], I must catch buses and a motorbike taxi. [Female 1, 70, Thanh Hoa, with COPD].*

*Money is not a problem for me. But honestly, my children’s work might be disrupted because of me. They take turns taking care of me. Some are working for the Government; some have their own business. I do not want them to resent me. (Male, 81, Hanoi, with COPD).*

### Ability to access care

In general, participants emphasised in their accounts that healthcare services were only sought when self-medication was unsuccessful, and the symptoms worsened. As most patients with CRD were older people, their access to healthcare was largely limited by the distance they could travel. Therefore, many participants chose closer health facilities instead of other hospitals whose healthcare services were perceived to be better. For example:

*In [that hospital], their lung treatment was so good that [my dad] felt much better after taking their medicines. Here, his condition has improved more slowly, but as [that hospital] is so far away we come here instead. (Daughter of a 73-year-old COPD male participant, Ca Mau).*

Support by patients’ families, such as from a family member who could accompany the participants to the health facilities, also affected their ability to reach the healthcare services. When family support was available, their access to health care was perceived to be easier:

*I felt tired, so I told my friend to call my son. Let us go to [that hospital]. So, I gave him my son‘s number then my son took me there by his motorbike. (Male, 53, Hanoi, with COPD).*

### Ability to pay for treatment

Without an effective management plan, patients with CRDs could experience frequent exacerbations, which leads to worsening of their symptoms as well as increasing healthcare costs. Patients with health insurance was perceived to help reduce the out-of-pocket costs for medical services. For example:

*This health insurance is voluntary. It costs around 600 [thousand VND] (≈US$25) a year. But it actually helps. Without it, I could not have afforded [the treatment]. I only pay less than 200 [thousand VND] (≈US$9) because the insurance covers 80% [of the total cost]. It helps so much. (Male, 71, HCMC, COPD).*

For some participants of particular groups such as the elderly or wounded soldiers, the health insurance could even provide them with privileges. These included the ability to access health facilities at higher levels without requiring a referral from the local health facilities, or covering all treatment costs, as indicated below:

*This is the hospital of highest level in the province. All other hospitals will refer patients to this hospital. I only can come here because I have the insurance for wounded soldiers. (Male, 69, Ca Mau, COPD).*

While health insurance covered some costs, for several patients the remaining cost was still a financial burden and unaffordable. This burden was also aggravated when prescribed medications were not covered by insurance; prescriptions that were often considered by clinical personnel to be inappropriate for the condition. Thus, they chose not to purchase some prescribed medications, although they were not necessarily aware that these prescriptions were not necessary.

*I have a voluntary health insurance. If I go to this hospital, the insurance will cover 80% [of the costs] and I will have to pay 20%. Extra medications cost 1.5 million (≈US$65). I could not buy some prescriptions because I ran out of money. I cannot tell the doctors that either. (Male, 64, Hanoi, COPD).*

When out-of-pocket payment was impossible due to their financial circumstances, the participants would choose to remain in the health facility whose services were more affordable, instead of going to another hospital which was perceived to have higher quality treatment for their conditions. For example:

*Half of the month, or a month after I go back home, I would feel tired again, it does not go away so I do not like that hospital. People told me to go to other places, but I cannot afford it. You know, like hospitals in HCMC where they have specialised hospitals, and my condition can be treated completely but I cannot afford to go there. (Male, 72, Ca Mau, COPD).*

As indicated above, there is a common perception among the participants that higher-level health facilities will provide a better quality of service. Thus, several participants chose to pay out-of-pocket to go to these hospitals, despite the financial burden.

*It has cost a great deal [since I came here], including medication costs, food cost,* etc. *My nephew just told me it had cost around 20 million (≈US$860) already. It is complicated as we are farmers, so we depend on the fields, but my wife and I are both here. We have a grocery store, but it has been closed for two months now since I was sick. Still, my family told me not to go to other hospitals, just choose [this hospital]. (Male, 45, Others, COPD).*

### Ability to engage with long-term care

Treatment and management of CRDs are long-term and require good adherence to a symptom management plan. The ability of participants to adhere to their long-term management plan was affected by perceived quality of healthcare services and attitudes of health workers. Patients with positive experience were more likely to stay in the long-term care.

*I have gone to many hospitals, but I see [this hospital] is good. First, the rooms are spacious and clean. The doctors are friendly and thoughtful. When I come here to obtain medications, they instruct me how to use the spray. I honestly think it is wonderful here, from their attitude towards patients to the hospital’s facilities. (Male, 60, Thanh Hoa, COPD).*

Support from patients’ families was also regarded as another factor influencing patient engagement. As mentioned in the excerpts above on patients’ ‘Ability to seek care’ and the ‘Ability to access care’, those who received support from their family could also better adhere to health workers’ instructions.

*My wife helps me with the treatment at home. She took care of the nebulizer and the inhaler and other medications. She reminds me regularly. (Male, 72, Thanh Hoa, COPD).*

However, some study participants frequently revealed having unpleasant experiences when seeking healthcare at certain health facilities, leading to interruption or changes in their management plan.

*When I came to the hospital [provincial hospital], they said “Well, this disease cannot be treated, just wait to die”. I will never come back that hospital. Then I came to [national hospital], they explained clearly my disease. It was so different. That’s why I come here, although it takes time and money (Male, 74, Ca Mau, COPD).*

*My wife and I were in [district hospital] and I was not happy. I asked them why my wife was so tired, and they shouted at me. Then, I don’t want to come back there anymore. So, I choose this [hospital] (Male, 72, Ca Mau, COPD).*

While long-term care requires patients to visit hospital regularly to get medication, not all patients could afford such regular visit, particularly if they choose hospital at higher level which often is far from their home.

*I did not trust the diagnosis of this hospital [provincial hospital] so I went to city hospital [Ho Chi Minh city]. Doctor asked me to return so many times, every month. Occasionally he allowed me to return after two months when I was busy. It costs me so much so I returned to this provincial hospital for treatment. (Female, 57, Ca Mau, asthma).*

## Discussion

This study identified complex, multi-directional pathways to healthcare in Vietnamese patients with chronic respiratory conditions. Initial treatment typically started with self-medication when their symptoms first appeared. This stage may be prolonged, until symptoms are no longer bearable without additional intervention. Most participants presented to several health facilities of different levels until their diagnoses were confirmed, usually at provincial- or national-level hospitals. Applying the model from Levesque et al. (2013) on access to care, we identified important factors that affected patients’ access to healthcare. These include health literacy and the availability of social support, which within the Levesque framework may be termed *abilities* and the factors which would be considered to relate more directly to the *system’s accessibility* patient’s trust in the quality of healthcare, financial burden of healthcare, health system factors (including the coverage of public health insurance, the distance to health facility, and attitudes of healthcare providers).

In terms of abilities self-medication is a common health behaviour for Vietnamese people [30, 31]. People often seek health advice from family members, friends or acquaintances and obtain medicines from local community pharmacies without prescription [32]. Often, it is only when self-medication cannot improve their conditions that formal health services will be sought, which can consequently delay the diagnosis and timely treatment of the diseases. Self-medication was also indicated as a common practice among diagnosed patients in this study when their symptoms were under control. Previous studies have showed that self-medication that included the use of drugs and traditional herbal medicines are still important elements of healthcare in Vietnam [30, 32–34]. Patients with respiratory symptoms can easily buy antibiotics in community pharmacies without prescription [35–37]. Such behaviour may result from inadequate health literacy among patients, especially about the harmfulness of self-prescription and the risk of delayed diagnosis and treatment for their conditions.

Indeed, participants lacked insight into their conditions owing to the limited communication provided by healthcare workers within both lower and higher levels of the healthcare system. Although patients understood they had received a diagnosis of a lung and respiratory disease that they may even do not remember the names, many were not aware that their condition was unlikely to be cured and may require long-term treatment to prevent future exacerbations. As a result, management of their respiratory illness frequently relied upon inappropriate self-medication for acute symptoms, instead of seeking preventive interventions or attending healthcare services to obtain appropriate treatment. Adequate awareness of their conditions is critical for CRD patients [38]. Previous research has shown that the lack of knowledge about CRD negatively affected decision-making about whether to participate in beneficial therapy such as pulmonary rehabilitation [39]. Additionally, findings from this study suggest that inadequate awareness of the conditions might also lead to poor adherence to treatment. Widely practiced self-medication, and the insufficiency of patients’ understanding about their condition, even among those with a formal diagnosis of CRD, highlighted the importance of integrating counselling or consulting sessions into health professionals’ practice. Future interventions are required to facilitate the timely access of patients with respiratory symptoms to expert clinicians, and to strengthen education that will improve treatment adherence [40]. The need for health education providing information about the patients’ conditions as well as appropriate management was also expressed by CRD patients themselves in previous studies [29, 41, 42]. Furthermore, interventions involving pharmacists to encourage customers to seek appropriate medical attention if their symptoms persists could help to improve the linkages between patients and the health system [43].

Support from patients’ social networks, especially support from family members, is another important factor that affected their access to care, especially for older patients. Specifically, the degree of family support affected their ability to seek and reach healthcare services. This finding aligns with previous research on patients with CRD in developed countries that found that having a caregiver within the family could significantly increase the possibility of accessing pulmonary rehabilitation, in comparison to those without a caregiver [39, 44]. Moreover, social support, whether from family members or friends, has also been shown in several systematic reviews to facilitate better treatment adherence in CRD patients, by providing regular reminders to take their medication on time [40, 41, 45, 46]. The findings from the current study and previous research have suggested potential interventions aiming to improve CRD treatment by targeting caregivers, such as through health education interventions.

The capacity of healthcare providers in the lower levels of the healthcare system was a particularly important determinant of patients’ access to timely diagnosis. The skills and attitude of clinicians were regarded as important factors to determine patients’ trust, willingness to attend health facilities and adherence to long-term therapy. This finding was consistent with results of previous research, which indicated that effective patient-health professional relationships where patients perceived healthcare providers as showing compassion and care can positively influence patients’ adherence for treatment of CRD [40, 47]. The connection between patients and healthcare providers can also be strengthened by improving communication skills for health professionals. Healthcare providers with improved communication skills can then provide more effective health education sessions for patients and at the same time, create and enhance the patients’ trust towards healthcare providers [38, 40, 48, 49]. Although the framework supports the identification of influential factors related to individual abilities and the system’s accessibility, distinguishing them into two categories may inadvertently conceal the interaction between the two: individual ‘abilities’ are substantially shaped by the ‘system’ and its weaknesses, rather than the fault of individuals themselves. The solutions to address individual abilities and system accessibility are inter-connected. The critical point of intervention may be to invest more heavily *in* making the system more accessible to address the ability of individuals to engage *with* the system. For example, the health system needs to engage in educating communities to address individual health literacy in order to expedite timely engagement in seeking care and reducing individual’s reliance on self-medication.

Treatment costs can be a burden to patients as demonstrated in the current study and the literature [42, 46, 50]. For this reason, Vietnamese public health insurance which covers at least 80% up to 100% of service and medication costs helped to increase the ability of patients with CRD to afford care. However, 13% of the population in Vietnam is still not covered by health insurance [51]. Additionally, health insurance rebates do not yet cover all medications and healthcare services for patients with chronic respiratory conditions. Updating the scope of health insurance coverage for patients with CRD could address this limitation to patient care.

Although the Levesque et al. (2013)‘s model identified relevant factors affecting healthcare seeking pathways of COPD and asthma patients, it could not explain complex reasons for patients’ progress through the multi-directional pathways. Patients do not necessarily move sequentially in a linear fashion through the care pathway. Given the challenges in receiving a diagnosis and then the need for long-term management of these chronic conditions, we found that the process was more commonly non-linear, involving loops and cycles. During the long-term health care process, patients were not only pushed forward but also pushed back to access health care at various health care facilities. This progress is substantially mediated by patients’ knowledge; therefore, strengthening the opportunities for improving patients’ knowledge about their disease and long-term care plan is very important as a means to expedite patients entry into and sustained engagement in efficient and effective care. Further, there needs to be additional support, which is sensitive to addressing gendered and societal norms, which impede individuals’ sustained engagement in care, especially during periods where hospitalization is necessary to provide effective care.

The study has several limitations. First, as we aimed to focus on patients’ perspectives, we did not explore the perspectives of healthcare providers regarding the facilitators and barriers of access to effective healthcare. Comparison between health workers’ and patients’ perceptions was, therefore, not possible. Second, although we tried to achieve a range of the participants’ characteristics, the majority of the respondents were men – reflecting the predominant population affected by smoking-related lung disease in Vietnam. Thus, we have not fully explored different factors affecting the healthcare seeking behaviours of women with chronic respiratory illnesses. Finally, as only provincial- and national-level hospitals were capable of definitively diagnosing COPD and asthma, we were not able to recruit patients from primary care health facilities, since few patients had definitive diagnoses in those settings. Despite these limitations, as we were able to recruit a relatively balanced number of participants from four study provinces, supporting the generalizability of our findings. We believe that the findings of this study that can strengthen design of clinical care pathways and future research to ensure that patient perspectives remain central to future healthcare reforms.

## Conclusions

In conclusion, Vietnamese patients with CRD had to navigate multiple encounters with healthcare providers before being appropriately diagnosed and treated. Access to healthcare was affected considerably by participants’ limited knowledge of their respiratory conditions, the availability of social support, especially from family members, as well as health system factors (mainly public health insurance coverage and the attitudes of healthcare providers). The study demonstrates the needs for health education interventions or programmes for both patients and their caregivers to improve timely diagnosis and effective management of respiratory disease. Interventions that can improve patient care include strengthening the communication skills of health workers and decentralisation of services to diagnose and treat chronic lung disease at the lower levels of the healthcare system. These approaches promise to make a major contribution to control of chronic lung diseases in Vietnam.

## Data Availability

The datasets generated during and analysed during the current study are not publicly available due to confidentiality policies of the study and as consented by the participants but are available from the corresponding author on reasonable request.

## Abbreviations

ACOS: Asthma-COPD overlap syndrome
COPD: Chronic obstructive pulmonary disease
CRD: Chronic respiratory disease
LMICs: Low- and middle-income countries
PAL: Practical approach to Lung Health
CMU: Chronic lung disease Management Unit
HCMC: Ho Chi Minh City
